# The LHCb Stripping Project: Sustainable Legacy Data Processing for High-Energy Physics

**DOI:** 10.1007/s41781-025-00151-6

**Published:** 2025-11-28

**Authors:** Nathan Allen Grieser, Eduardo Rodrigues, Niladri Sahoo, Shuqi Sheng, Nicole Skidmore, Mark Smith

**Affiliations:** 1 University of Cincinnati, Cincinnati, USA; 2 Oliver Lodge Laboratory, University of Liverpool, Liverpool, UK; 3 University of Birmingham, Birmingham, UK; 4 École Polytechnique Fédérale de Lausanne, Lausanne, Switzerland; 5 Institute of High Energy Physics, Chinese Academy of Sciences, Beijing, China; 6 Department of Physics, University of Warwick, Coventry, UK; 7 Imperial College, London, UK

**Keywords:** High-energy physics, LHCb experiment, Data processing and offline analysis

## Abstract

The LHCb Stripping project is a pivotal component of the experiment’s data processing framework, designed to refine vast volumes of collision data into manageable samples for offline analysis. It ensures the re-analysis of Runs 1 and 2 legacy data, maintains the software stack, and executes (re-)Stripping campaigns. As the focus shifts toward newer data sets, the project continues to optimize infrastructure for both legacy and live data processing. This paper provides a comprehensive overview of the Stripping framework, detailing its Python-configurable architecture, integration with LHCb computing systems, and large-scale campaign management. We highlight organizational advancements, such as GitLab-based workflows, continuous integration, automation, and parallelized processing, alongside computational challenges. Finally, we discuss lessons learned and outline a future road-map to sustain efficient access to valuable physics legacy data sets for the LHCb collaboration.

## Introduction

The LHCb Stripping project [1] plays a vital role in filtering the experiment’s data, serving as the last central offline processing step, where physicists select interesting particle interactions from billions of recorded events. Its flexible Python interface allows researchers to customize selection criteria for different physics studies.

Integrated into the LHCb Data Processing & Analysis (DPA) [2] framework since 2020, the Stripping project is a central pillar of DPA’s Work Package 5 (WP5) [3], which focuses on legacy software and data. WP5 ensures that legacy data collected at CERN’s Large Hadron Collider (LHC) [4, 5] during Runs 1 (2010–2012) and 2 (2015–2018) remain accessible for future re-analysis by maintaining the legacy software stack and organizing necessary (re-)Stripping campaigns. These campaigns, which ran concurrently with data-taking or during End-of-Year (EoY) periods, involve skimming and slimming data to extract the most relevant information for physics analysis.

The Stripping project is designed to streamline the raw data into a manageable subset for offline analysis that maximize signal retention while minimizing background. Its Python-configurable architecture allows for flexible and user-oriented data selection, while modern organizational tools, such as GitLab Milestones, are employed to track developments and ensure timely completion of tasks.

## LHCb Dataflow and Stripping Software Framework

The LHCb data processing model spans multiple stages, from initial triggering to offline data selection. Stripping reduces the volume of recorded data by selecting events of interest and saving only the necessary information for physics analysis, producing smaller, more manageable data sets. The Stripping software stack [1] integrates closely with the DaVinci [6] framework. DaVinci is an offline analysis package which performs the event-by-event processing to produce user-level nTuples, and harmonizes selection tools across the Stripping stage and the end-user stage.

**Fig. 1:**
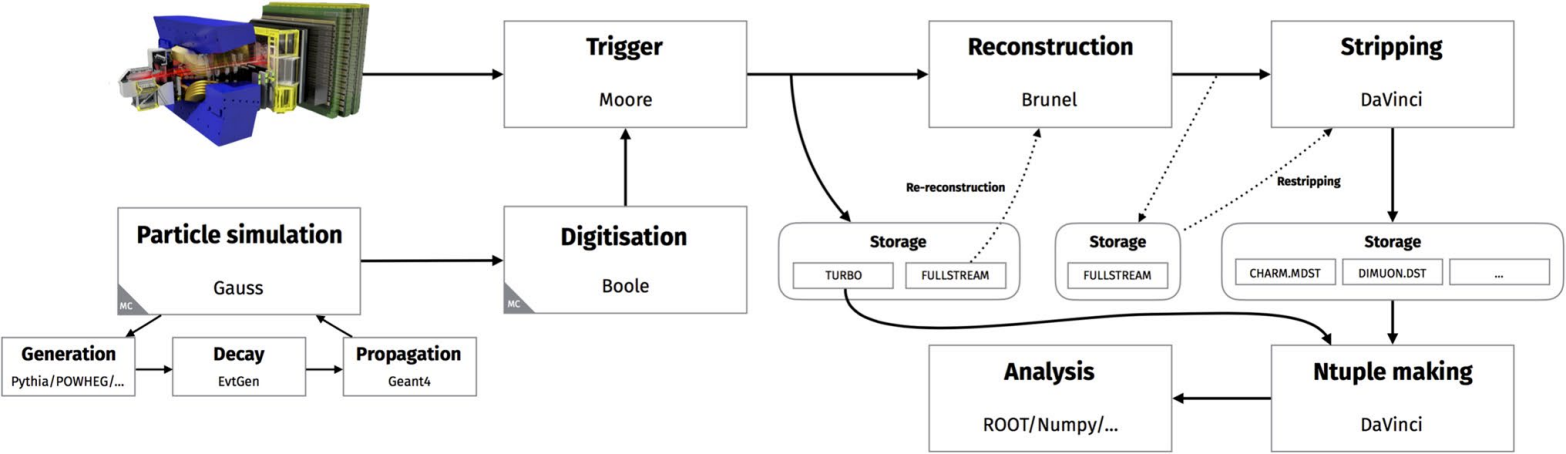
LHCb dataflow in Run 2 [13] as described in "LHCb Dataflow and Stripping Software Framework" Section. The (re-)Stripping stage serves as the last centralized production stage before offline analysis workflows

The LHC provides proton–proton collisions to the LHCb experiment at an interaction rate of 40 MHz, generating an enormous volume of data–approximately 1 TB every second in Run 2. Storing all this data is impractical due to cost constraints, necessitating a sophisticated filtering process to retain only the most interesting events, as shown in Fig. 1. This raises critical challenges: how to process and filter data quickly and accurately, manage complex tasks, organize collision data flexibly, and configure software without recompilation. These challenges stem from the high data arrival rate and the complexity of the data, which contains thousands of potential decay combinations of interest. To address these issues, the LHCb experiment employs a multi-step data processing chain. The Run 1+2 data flow begins with the trigger system, which filters events using hardware (Level-0, L0) and software (High Level Trigger, HLT) components [7]. The triggered data are then reconstructed by the Brunel application [8], transforming raw detector hits into tracks and particle identification information stored in DST (Data Summary Tape) 1 files, allowing candidates (often a particle and its decay products) selected by the HLT to be saved directly to disk, with selections triggered by identifying specific particle types passing a specific set of kinematic requirements. Further filtering is performed through the Stripping process, managed by the DaVinci application, which outputs data in DST or $\upmu$DST (micro-DST) formats, grouped into physics-specific streams to optimize storage and analysis efficiency.

Simulated data are processed through an identical reconstruction chain as real data, maintaining consistent treatment of detector effects and systematic uncertainties.

The simulation begins with the Gauss [9] application, which models proton–proton collisions and particle decays using generators, such as Pythia [10] and EvtGen [11], followed by Geant4 [12] for detector response simulation. The Boole [9] application converts simulated hits into signals mimicking real detector output, allowing the simulated data to be processed through the same reconstruction and Stripping steps as data.

The LHCb software is modular, with each application (e.g., Brunel, DaVinci) handling specific tasks, enabling flexibility and efficiency in data processing. Stripping campaigns, identified by versions like SXrYpZ, are central to defining the available physical processes for analysis. Major Stripping versions (X) represent full reprocessing and correspond to data set years. Minor versions (Y) correspond to superseding full reprocessings, while patch versions (Z) indicate incremental updates. Understanding the reconstruction and Stripping versions is crucial for selecting data, as these versions significantly impact the physics results. The Stripping project website provides detailed information on Stripping configurations, algorithms, and selection criteria, serving as essential resources for analysts.

The Stripping project is maintained across multiple Git production branches, each tailored to specific data-taking periods and campaign types. These branches are designed to support legacy data processing while incorporating necessary bug fixes and updates. New releases are prepared following the completion of development and validation phases, facilitated by an in-depth testing infrastructure.

**Fig. 2:**
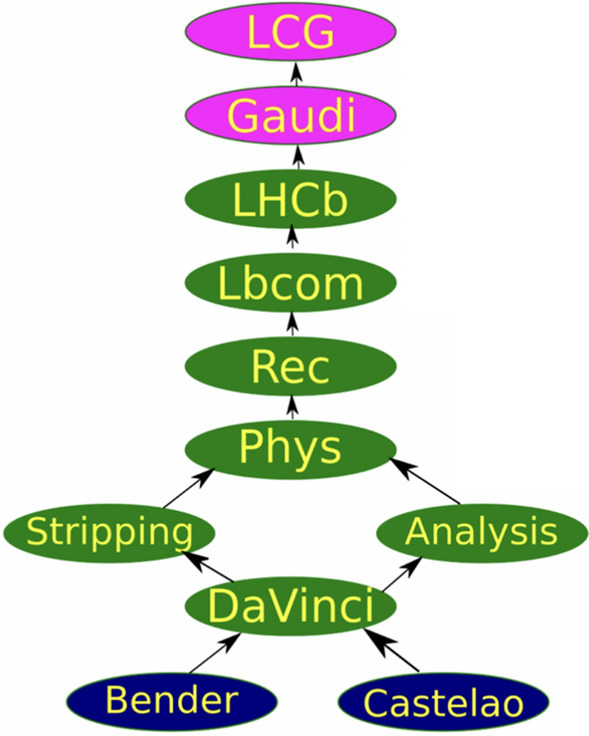
Schematic of the legacy software stack utilized for the LHCb offline data processing stage during Runs 1 and 2

The LHCb Stripping software project is operating atop the primary analysis software, DaVinci, which is built on the Gaudi [14] and LHCb frameworks. The Stripping package, which defines all selection lines and their configurations, is part of a larger software stack, as shown in Fig. 2, that includes dependencies such as Phys [15], Rec [16], LbCom [17], and LHCb [18]. The Analysis project is a complimentary sub-project of DaVinci that contains additional tools for the analysis-level use of DaVinci that are not utilized in the Stripping processing stage. This stack relies on CERN’s LHC Computing Grid (LCG) software distribution [19], with specific ROOT versions determined by the LCG release.

The LHCb Stripping project performs the crucial first stage of offline data processing, transforming reconstructed collision data into manageable subsets for physics analysis. Using a Python-configurable architecture, it enables analysts to select specific physics candidates from the processed data sets, which are stored as 5 GB files on both disk and tape (with placement optimized for storage availability). When new physics selections are developed or existing ones require improved performance, the collaboration conducts systematic “(Re-)Stripping campaigns" – comprehensive reprocessing efforts that apply updated selection criteria to the full legacy data sets, ensuring consistent analysis selections across LHCb’s physics program.

Stripping campaigns are large-scale efforts that involve collaboration-wide discussions, preparation of selection lines by analysts and Physics Working Groups (PWGs), software stack preparation, validation through mini-productions, and the final production of data sets. These campaigns are categorized into three types: prompt campaigns during data-taking (now obsolete), full campaigns, and incremental campaigns. Full campaigns process all available selection lines, while incremental campaigns focus on newly added or modified lines.

The campaigns are coordinated by Stripping coordinators, PWG liaisons, analysts, and the Distributed Computing team, with planning and execution spanning several months. The Stripping project’s evolution has seen the consolidation of legacy production branches to reduce maintenance overhead. The project’s structure and workflow ensure the preservation of legacy data while supporting ongoing and future physics analyses, with detailed documentation and release schedules provided to facilitate collaboration-wide coordination.

## Stripping Campaign Workflow and Organization

The streamlined workflow outlined in this section represents the optimized framework refined during the 2023–2024 incremental re-Stripping campaign, incorporating significant enhancements over previous iterations. This current framework establishes a flexible baseline for future campaigns, facilitating integration of modern and evolving toolkits.

This reprocessing initiative was launched in response to analysis requirements. A pre-campaign needs assessment revealed that most Physics Working Groups (PWGs) primarily aimed to improve ongoing analyses through updated selection criteria.

### Roles and Responsibilities in the Stripping Campaign

**Fig. 3:**
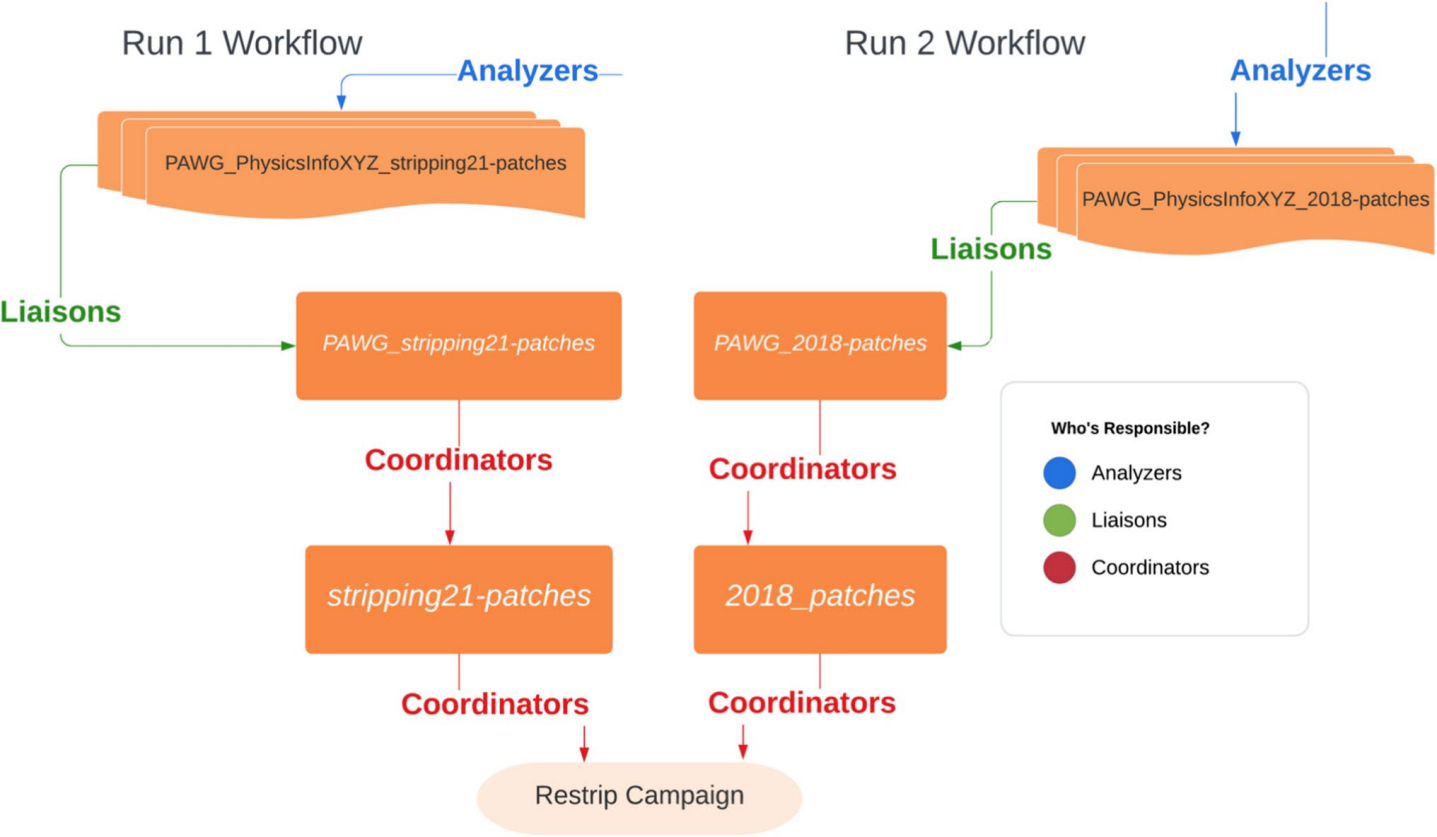
GitLab workflow for the development of the recent Stripping campaign. The workflow is compartmentalized to allow significant reductions of top-level review while allowing lower level reviews to have a closer focus on the physics performance

The central Stripping coordination team serve as the interface between Operations and Physics Planning Groups. Their responsibilities encompassed preparing testing infrastructure (repositories, data files, and DaVinci caches), establishing validation procedures, reviewing and accepted GitLab Merge Requests (MRs),^2^ implementing nightly tests for DaVinci and Stripping projects, and ultimately managing production requests. They also maintained campaign documentation and workflow oversight, as illustrated in Fig. 3.

Each PWG appoints liaisons to facilitate the Stripping campaign. Their key duties included disseminating critical Stripping information (deadlines, workflows, documentation) to their PWG members, assisting with line writing and validation, and monitoring MRs to ensure successful Continuous Integration(CI) tests, an automated process that runs tests on code changes whenever they are pushed to a GitLab repository. Liaisons were specifically responsible for testing in two core packages: (1) Phys/StrippingSelections, verifying line rates and timing constraints, and (2) Phys/StrippingSettings, preparing and validating PWG-wide configuration files before developer handoff.

### Stripping Campaign Methodology

The re-Stripping campaign follows a well-defined workflow comprising three key phases: line development, validation testing, and final production approval. While these campaigns have traditionally operated as large-scale coordinated efforts with strict timelines, the latest campaign marked a significant transition in execution procedure, with significant changes to the workflow and management procedures.

#### Development, Code Review and Merge Request Workflow

During the Stripping campaign, analysts develop modules in their respective packages and submit MRs to the PWG-specific development branch. Liaisons oversee these MRs by: (1) ensuring proper naming conventions, correct branch assignments, and linkage to relevant GitLab Milestones and Issues for tracking; (2) validating that Stripping lines meet performance thresholds ($\leqslant$0.05% rate for DST, $\leqslant$0.5% for $\upmu$DST, and $\leqslant$ 1 ms per event timing); and (3) confirming CI tests pass before merging. GitLab’s CI system ensures code compatibility by running functional tests on physics selections, monitoring algorithm rates and timing, and organizing PWG contributions efficiently. These CI tests perform linting and execute data set-specific tests for each year’s data. In addition, to reduce operational overhead, CI tests are defined to only run specific working groups’ lines on the related development branches. After approval, liaisons prepare line dictionaries, enabling flexible Stripping campaign configuration per version, for final review and ensure all PWG branches are merged into the production branch by the following week to maintain the validation schedule.

#### Validation Phase and Performance Verification

Following the development stage, a dedicated testing and validation phase, conducted in close collaboration with PWG liaisons, ensures selections and physics performance meet expectations. Analysts use larger data sets, produced using the official, central production workflow, to identify bugs or unintended selection effects, following the same workflow as the development stage. To maintain the schedule, no new selection lines may be introduced during validation.

#### YAML-Based Production Management

The Stripping production workflow has been modernized through the transition from JIRA [20] to GitLab for coordinator–production team communication, in alignment with Run-3 offline production standards. The new workflow implements YAML configuration files to manage Dirac [21] requests, containing complete processing parameters (including years, conditions, data packages and applications) for improved re-usability. Notably, when a campaign’s validation phase is completed ahead of schedule, opportunities to further refine the production request process are able to be included in the overall campaign workflow.

### Event Metrics in Stripping Campaigns

This section presents a detailed analysis of Stripped events, comparing per-stream and globally averaged metrics across recent and past re-Stripping campaigns.

The latest incremental re-Stripping of Run 1 (2.5 PB input) reduced the output to 350 TB, achieving a size reduction factor of 7. A 2023–2024 incremental campaign on 6.17 PB of 2016–2018 data produced 1.48 PB, reducing the total size by a factor of 4.2. Further details on event counts, files, and stream-specific storage allocations are provided in Table 1. For Run 2, incremental campaigns consistently yield smaller outputs (by a factor of $\geqslant$2) compared to full re-Stripping due to selective processing.

**Table 1:** Sample sizes in TB for the latest incremental re-Stripping campaigns (except the 2015 data set, which has not received an incremental re-Stripping following its latest full re-Stripping)

Year	Processing	Total size (RAW)	Size out	Reduction factor
2011	Stripping21r1p2	644.0	78.2	8.2
2012	Stripping21r0p2	1795.2	271.3	6.6
2015	Stripping24r2	882.2	161.1	5.5
2016	Stripping28r2p2	2657.0	476.7	5.6
2017	Stripping29r2p3	2276.0	444.6	5.1
2018	Stripping34r0p3	2754.0	557.8	4.9

The “Processing” is the version of campaign. The “RAW” and “out” columns refer to the input and output samples, both in units of TB

Table 2 provides a yearly breakdown, categorized by magnet polarity, of the Stripping campaigns summarized in Table 1. It details the average per-event sizes of the input and output samples as well as the reduction factor in the total number (the results were averaged across all streams, with each stream specifically optimized for different physics analyses as outlined later) of Stripped events. There is broad agreement of all quantities irrespective of the magnet polarity. Events selected in the 2012 samples are 10% larger than events selected in the 2011 samples, which reflects a higher event multiplicity given the larger beam energy in 2012 (4.0 TeV) compared to 2011 (3.5 TeV).

**Table 2:** Average per-event sizes in kB for the latest re-Stripping campaigns

Year	Processing	Polarity	Avg kB/evt (in)	Avg kB/evt (out)	Reduction rate
2011	Stripping21r1p2	Down	58.3	30.2	7.9
		Up	58.3	30.1	8.7
2012	Stripping21r0p2	Down	64.0	34.0	6.8
		Up	65.0	34.0	6.5
2015	Stripping24r2	Down	39.0	29.7	3.9
		Up	37.1	29.9	4.0
2016	Stripping28r2p2	Down	49.6	32.3	4.3
		Up	48.1	32.7	4.3
2017	Stripping29r2p3	Down	53.9	31.2	4.2
		Up	56.2	31.2	4.4
2018	Stripping34r0p3	Down	55.8	31.2	3.9
		Up	55.3	31.0	4.0

The “Reduction rate” indicates the reduction in the total size of the samples Stripped

For all Run-2 campaigns considered, the average event sizes are more homogeneous in a given campaign — as can be seen by comparing, for example, the latest campaign Stripping34r0p3 (2018), Stripping29r2p3 (2017) and Stripping28r2p2 (2016) — given that the beam energy was consistently 6.5 TeV for proton–proton (*pp*) collisions. Average (over all output streams) Run-2 Stripped event sizes are around 25–35 kB, similar to the event sizes for Run 1. Large differences in the ratio of events selected by the Stripping and the total sample size reduction are observed among years but most significantly between incremental and full re-Stripping campaigns, again according to expectations due to trigger evolution during Run 2. For full campaigns the fraction of selected events (over all streams) is often as high as 70%, a number that seems rather large. This illustrates that slimming (removing unneeded object information) events — rather than skimming (removing entirely) events — can result in larger reductions of output data from the Stripping process. Typical total sample size reductions for incremental and full re-Stripping campaigns are 4–5 and 2–3, respectively.

Output streams are systematically defined for both Run-1 and Run-2 campaigns as below:

1. BHADRON.MDST
2. BHADRONCOMPLETEEVENT.DST
3. CHARM.MDST
4. CHARMCOMPLETEEVENT.DST
5. DIMUON.DST
6. EW.DST
7. LEPTONIC.MDST
8. SEMILEPTONIC.DST

The majority of campaigns utilized between 7 and 9 distinct output streams, with many specifically designed for individual PWGs—such as EW.DST and SEMILEPTONIC.DST—while others accommodated more comprehensive physics analyses involving multiple PWGs. Specialized streams include CALIBRATION.DST for detector calibration and FTAG.DST for flavor tagging.

**Fig. 4:**
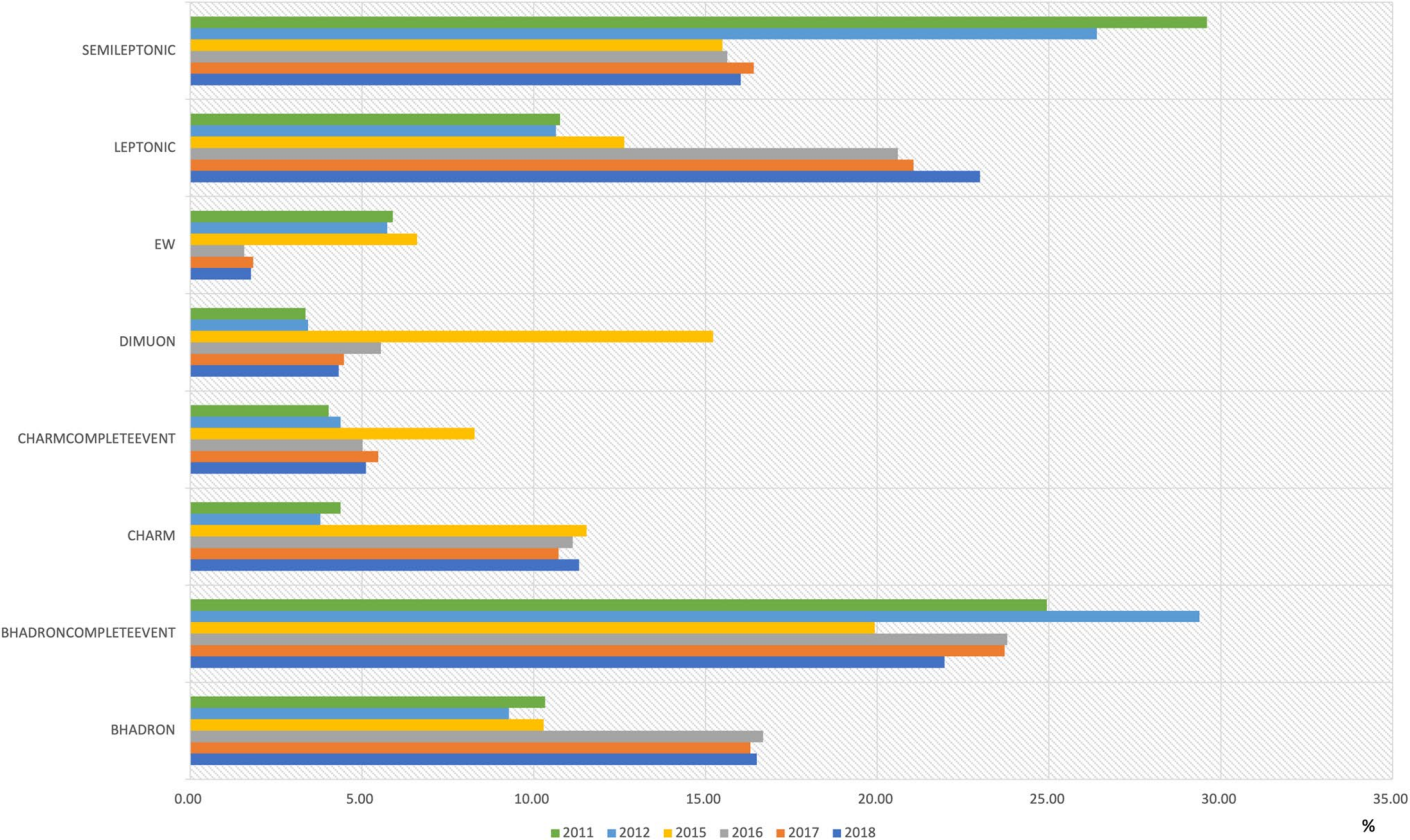
Share of storage space taken by the various output streams in Table 1. Values are provided as percentages out of 100

Figure 4 illustrates the relative storage space allocation across different output streams. The observed distribution of output sample sizes for the latest Run-1 and Run-2 incremental campaigns (detailed in Table 1) is consistent with expectations, as essentially identical selection criteria were applied across all campaign years. Notably, the BHADRONCOMPLETEEVENT.DST, LEPTONIC.MDST, and SEMILEPTONIC.DST streams collectively account for approximately two-thirds of the total output volume.

**Fig. 5:**
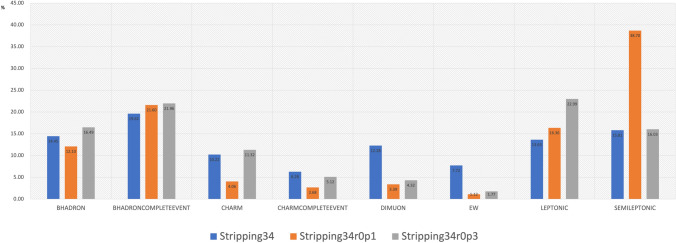
Share of storage space taken by the various output streams in two Run-2 2018 campaigns. Values are provided as percentages out of 100

For the 2018 data set, Fig. 5 shows the comparison between the full Stripping34 campaign and various incremental re-Stripping campaigns. Large differences can be observed in the shares taken by the various output streams. Though a detailed comparison is non-trivial and of limited interest, it is a fact that the differences directly reflect the sets of selections included by the various PWGs in any given campaign.

Table 3 details the number of Stripped events and, most importantly, the total output sizes (in TB) and average per-event sizes (in kB) for the DIMUON.DST stream that was produced by any of the considered campaigns. Wide differences in average event size are seen across the streams. For example, a typical event size in the DST output streams (rather than $\upmu$DST) is 80–100 kB — here, streams persist selected events in full, which analysts can further process downstream. In comparison, events in “standard" streams, e.g., the BHADRON.MDST stream, require as little as 10–15 kB, though average sizes up to 50 kB are also seen, in particular for full campaigns. The SEMILEPTONIC.DST stream, whereby partially reconstructed events must be selected, lies somewhat in the middle, with average event sizes around 70–80 kB. The average event sizes depend little on whether a campaign is incremental or not, as expected, since the average per-event size should not depend much on the set of lines run.

**Table 3:** Number of Stripped events, total storage sizes (in TB) and average per-event sizes for the DIMUON.DST stream for all campaigns compared

Year	Processing	Polarity	Events (out)	Total size (out)	Avg kB/evt (out)
2011	Stripping21r1p2	Down	19,674,496	1.6	80.0
		Up	13,437,108	1.1	78.7
2012	Stripping21r0p2	Down	49,560,939	4.7	93.9
		Up	49,268,945	4.6	94.2
2015	Stripping24r2	Down	132,938,710	14.2	107.1
		Up	96,773,326	10.3	106.2
2016	Stripping28r2p2	Down	105,990,750	13.7	129.1
		Up	100,173,860	12.8	127.6
2017	Stripping29r2p3	Down	88,091,847	10.3	117.3
		Up	82,218,042	9.6	116.5
2018	Stripping34r0p3	Down	100,096,435	11.9	119.3
		Up	104,280,590	12.2	116.5

Analysis of the SMOG^3^ [22] heavy-ion data reveals atypical event size evolution, where final IFT.^4^DST outputs (35 kB/event) triple intermediate-stage sizes, contrasting with *pp* campaigns, while stripping maintains 110–130 TB total volume through a factor 3 reduction.

## Operational and Computing Aspects

**Fig. 6:**
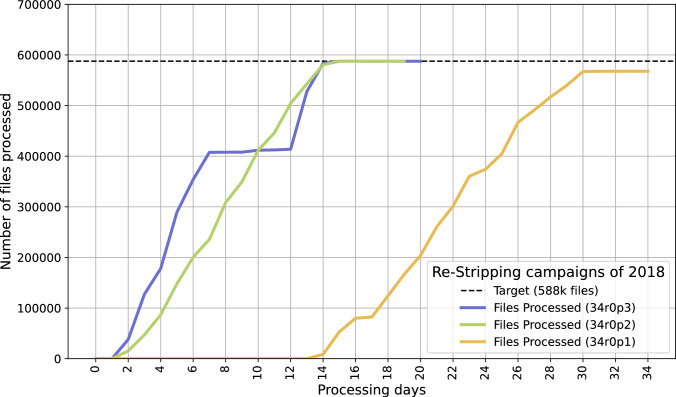
Comparison of several campaigns processing the 2018 data set, plotting versus the number of days of processing rather than the actual calendar dates. The curve for the campaign (Stripping)34r0p1 does not reach the "target line" as the other two campaigns, because, at that time, certain runs or files had been marked as unusable for physics based on data quality assessments. The curve does not otherwise reflect any operational issue. The multi-day pause in the middle of Stripping34r0p3 was due to planned resource unavailability from the distributed computing team, and is not considered in the overall production time comparison

Figure 6 shows the final three incremental re-Stripping campaigns performed on the 2018 data sample. Each campaign processing time showed improvement with each subsequent version, with the first reprocessing time taking 2 weeks, and the latest taking 1 week after removing scheduled operational pauses.

While legacy data software stacks often lag behind modern HEP developments, reprocessing older data with contemporary workflows can help meet experimental constraints. Analyses following major Stripping campaigns are crucial for procedural improvements, particularly given the high participant turnover in sporadic legacy productions. Targeted training programs have proven effective, as demonstrated by recent surveys showing enhanced participant confidence and professional development. Simultaneously, workflow optimization and progress monitoring are essential for maintaining operational efficiency in large reprocessing campaigns. Together, these human and technical factors enable more effective legacy data production while supporting researcher development.

## Summary

Building on the successful Run-3 Sprucing model, DPA WP5 proposes an iterative workflow for developing new Stripping campaigns for Runs 1 and 2 data. The approach features: (1) early line development when physics ideas emerge, (2) branch-based analyst work with CI testing, (3) regular PWG branch integration, (4) milestone tracking for resource planning, and (5) buffered deadlines for alignment with Physics Coordination and Operations Coordination priorities. This flexible system allows analysts the freedom to make developments when convenient while providing coordination teams evidence for campaign planning needs.

The LHCb collaboration maintains a vibrant legacy program using Runs 1 and 2 data sets through sustainable systems combining software development and large-scale reprocessing. The Stripping framework’s Python-based flexibility, combined with modern tools and strong computing and operations collaboration, manages campaign complexity while offering development opportunities for researchers at all career stages, even as focus shifts to Run 3 data.

## Data Availability

No data sets were generated or analysed during the current study.

## Footnotes

1 Files resulting from the reconstruction of real data and of MC samples, basically a ROOT file with our data in an optimized format.

2 Developers submit an MR to request that their changes be reviewed and merged into the target branch.

3 The LHCb System for Measuring the Overlap with Gas (SMOG) system enabled fixed-target proton-noble gas collisions during Run 2 by injecting a precise density of gas into the beam pipe intersection region.

4 IFT refers to the Ion and Fixed Target PWG of LHCb.
